# Towards standard methods for the classification of aquatic toxicity for biologically active household chemicals (BAHC) present in plastics, pharmaceuticals, and cosmetic products

**DOI:** 10.1007/s10661-021-09436-w

**Published:** 2021-10-02

**Authors:** Ricardo Beiras

**Affiliations:** 1 Department of Ecology and Animal Biology, Faculty of Marine Sciences, University of Vigo, 36310 Vigo, Galicia, Spain; 2 ECIMAT-CIM, University of Vigo, Illa de Toralla, 36331 Vigo, Galicia, Spain

**Keywords:** Water quality, Toxicity, Copepods, Echinoderms, Larvae, UV filters

## Abstract

A standard method to test the aquatic toxicity of biologically active household chemicals (BAHC), including those with very low water solubility, is proposed. The method uses the common marine models *Paracentrotus lividus* embryos and *Acartia clausi* larvae, in order to advance towards derivation of water quality criteria for these emerging pollutants that currently lack environmental standards. Depending on the water solubility and octanol–water partition coefficient (*K*_ow_) of the substance, the protocol consists of testing the toxicity of the substances by serial dilutions of water stocks, dimethyl-sulfoxide stocks, or 100 mg/L lixiviates in seawater. When this method is applied to eleven model BAHC, the pharmaceutical fluoxetine, the antioxidant butylated hydroxytoluene, and the UV filters broadly present in cosmetics octocrylene and 4-methylbenzylidene camphor, are classified as very toxic to aquatic life, since their EC_50_ values are < 1 mg/L. In general, both biological models, *P. lividus* and *A. clausi*, yield the same classification of the substances tested, but variations in the classification of aquatic toxicity depending on methodological aspects are discussed. The use of *A. clausi* nauplii provides more protecting value to the toxicity parameters obtained by using this protocol.

**Graphic abstract:**

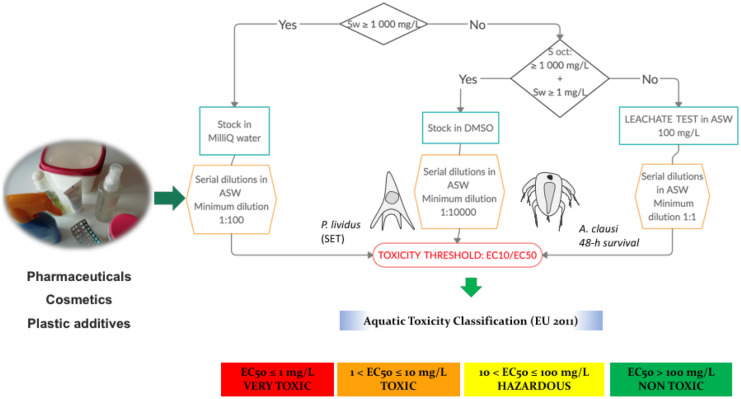

**Supplementary information:**

The online version contains supplementary material available at 10.1007/s10661-021-09436-w.

## Introduction

An increasing amount of synthetic chemicals are used to manufacture everyday items such as plastics, textiles, cosmetics, and pharmaceuticals, and for many of them, adverse biological effects on aquatic organisms have been reported [reviewed by Hahladakis et al. (2018) for plastic additives, Samchetshabam et al. (2017) for textiles, Beiras (2021) for cosmetics and pharmaceuticals]. These biologically active household chemicals (BAHC) reach surface waters through urban wastewater effluents and improper environmental disposal. Depuration in wastewater treatment plants rarely removes more than 90% of the chemical, and the remaining is discharged in the receiving estuarine and coastal waters. Even though most of these chemicals are non-persistent, in practice their continuous input to natural water bodies causes permanent exposure of organisms inhabiting receiving waters (Margot et al., 2015). Therefore, the frequent detection of BAHC in surface waters raises emerging concern on their potential ecotoxicological effects on coastal ecosystems (Beiras, 2018).

Plastics have become a ubiquitous feature of modern life, and their global production has risen to the current 359 million tons per year (Plastics Europe and Conversio Market & Strategy GmbH, 2020), around 50% for single-use disposable items. Plastics have two different components with very different toxicity and environmental fate: (i) the polymeric matrix consisting of long carbon chains with inert chemical properties, and (ii) a set of lower molecular weight chemicals, normally not bound to the polymer chains, termed functional additives, intentionally added to give the material the desired mechanical properties throughout the product’s life. The main plastic additives are plasticizers, flame retardants (e.g., tris 1-chloropropan-2-yl phosphate (TCPP), tris 1,3-dichloro-2-propyl phosphate (TDCP)), UV stabilizers (e.g., chimassorb, CHI), antioxidants (e.g., butylated hydroxytoluene, BHT), and antimicrobials (e.g., lawsone, LAW) (Bolgar et al., 2015; British Plastic Federation, 2021). Conventional polymers are produced by polymerization of olefins by petrochemical companies. These raw materials termed resins are later used for the fabrication of plastic products by adding the desired functional additives in a process termed compounding. Aquatic toxicity studies suggest low toxicity of the precompounding resins (Beiras et al., 2018; Cole et al., 2015; Kaposi et al., 2014; Vroom et al., 2017), whereas commercial plastic products frequently produce toxic leachates (Bejgarn et al., 2015; Lithner et al., 2009, 2012; Oliviero et al., 2019), pointing to additives as responsible for the toxicity. Toxicity on planktonic marine organisms of some plastic functional additives and cosmetic components at µg/L levels have been previously reported (Giraldo et al., 2017; Paredes et al., 2014; Tato et al., 2018).

The pharmaceutical industry produces approximately 3000 different synthetic chemicals including antibiotics, painkillers, antidepressants, adrenergic receptor blockers, antidiabetics, contraceptives, and lipid regulators, used to cure, prevent, or alleviate the symptoms of illness (Richardson, 2012). Most of them are used in such low amounts that they do not pose any significant environmental risk, but the most common ones, such as analgesics or psychiatric drugs, have an annual production within the order of 100 to 1000 Ton in Europe (Fent et al., 2006). Anti-inflammatory painkillers such as ibuprofen (IBU), antibiotics such as sulfamethoxazole (SMX), and antidepressants such as fluoxetine (FLU) have been reported to pose a significant environmental risk in aquatic environments (Beiras, 2021; Magureanu et al., 2015).

Personal care products such as shampoos, lotions, and skin-care creams include ingredients intended to inhibit microbial degradation (preservatives), UV filters such as cosmetics octocrylene (OCT) and 4-methylbenzylidene camphor (4-MBC) to prevent photo-oxidation, and fragrances such as galaxolide (HHCB) to provide good smell. In some cosmetics these components reach up to 30–40% of the final product. Sun screens are specifically used in recreational coastal areas, and swimmers represent a direct input for these substances into the water. Aromatic UV filters are highly lipophilic, with potential for bioaccumulation, and capable to cause endocrine disruption (Fent et al., 2008; Paredes et al., 2014). Maximum concentrations of UV filters in cosmetics in Europe are set in Regulation 1223/2009 (European Commission, 2009) (e.g., 4% for 4-MBC). In USA, the composition of cosmetics is regulated by (US FDA, 2018), and 4-MBC is not allowed as a sunscreen component.

While many synthetic pesticides and phytosanitary products are regulated and environmental standards are implemented (National acute and chronic water quality criteria from the US Environmental Protection Agency (US EPA, 2021), Canadian water quality guidelines (CCME, 2021), acute and chronic standards for priority pollutants in Directive 2013/39/EU, ANZECC guidelines in Australia and New Zealand (Warne et al., 2018)), BAHC are not included in any of these regulations. Ecotoxicological data obtained from toxicity tests conducted according to standard methods are needed in order to assess the ecological risk of these compounds in coastal environments and to set BAHC water quality criteria on solid scientific grounds. The aim of this study was to advance the standardization of methods intended to the classification of aquatic toxicity of BAHC, and to illustrate these methods with representative BAHC present in plastics, pharmaceuticals, and cosmetic products. In order to maximize the sensitivity of the tests, preference was given to early life stages (embryos and larvae) of the test species, *P. lividus* (embryos) and *A. clausi* (larvae) (Durán & Beiras, 2017).

## Materials and methods

### Test species

Test species were supplied by the Marine Biological Resources Service from ECIMAT (University of Vigo). Tests were conducted in compliance with internationally accepted standard methods. The *Paracentrotus lividus* sea-urchin embryo test (SET) followed Beiras et al. (2012), and the *Acartia* sp copepod 48-h survival followed ISO 14669 (ISO, 1999) using nauplius larvae. Briefly, *P. lividus* gametes were obtained by dissection from conditioned adults; eggs were carefully fertilized in a measuring cylinder adding a few µL of concentrated sperm while gently stirring with a plunger. Fertilization was assessed under a binocular microscope, and 40 fertilized eggs per mL were delivered for incubation into 4 mL glass vials with Teflon-lined caps. For copepods, < 24-h-old nauplii were separated from the adults by using a 40-µm metallic mesh and delivered one by one, employing a glass pipette under binocular stereoscope in 10 mL glass vials. A total of 10 individuals per vial were used. Endpoints recorded after 48 h were, for sea urchins, length (maximum dimension) in 35 individuals per vial using a Leica DMI 4000B inverted microscope, and for copepods nauplius larvae survival. Further details on the bioassay methods were provided by Tato et al. (2018) and Beiras et al. (2019). All experiments were conducted in isothermal rooms at 20 ± 0.5 °C.

### Experimental solutions

Toxicity tests were carried out using artificial seawater (ASW) with a defined chemical composition (Lorenzo et al., 2002) and oceanic characteristics (34 ± 2 psu salinity, 8.2 ± 0.1 pH, 8.0 ± 0.2 mg/L dissolved oxygen). OCT (CAS number: 6197-30-4), BHT (CAS number: 128-37-0), HHCB (CAS number: 122-05-5), 4-MBC (CAS number: 36861-47-9), TDCP (CAS number: 13674-87-8), IBU (CAS number: 15687-27-1), SMX (CAS number: 723-46-6), LAW (CAS number: 83-72-7), TCPP (CAS number: 13674-84-5), and FLU (CAS number: 56296-78-7) were purchased from Sigma-Aldrich (Milwaukee, WI, USA) and Merck (Darmstadt, Germany). CHI (CAS number: 192268-64-7) was obtained from AIMPLAS Technological Institute of Plastics (Valencia, Spain).

Based on the water and octanol solubility data of the substances used in this work (Table 1), we propose a decision tree to choose the best available methods for dosage of the chemicals reflected in Fig. S1. For chemicals with a water solubility (*S*_w_) ≥ 1000 mg/L, stocks were made up in Milli-Q® ultrapure water, and for those with *S*_w_ < 1000 mg/L and solubility in octanol ≥ 1000 mg/L stocks were made up in dimethyl-sulfoxide (DMSO). In both cases, serial dilutions of the stock in ASW were tested, up to a final concentration of at least 1 mg/L. Final DMSO concentration in experimental treatments was always below NOEC for both test species: 8.8 g/L for *P. lividus*, and 0.4 g/L for *A. clausi* (Tato et al., 2018).

**Table 1:** Toxicity of BAHC used in plastics, cosmetics, and pharmaceuticals on *Paracentrotus lividus* sea-urchin embryos

Chemical	CAS-Number	Use	Water solubility (mg/L)	Log *K*_*ow*_	Test	SET		Acute aquatic toxicity (EC/1272/2008)
						LOEC (µg/L)	EC_10_–CI 95% (µg/L)	
Fluoxetin (FLU)	56296-78-7	Pharmaceutical	4000	4.05	Water stock	30	14 (6–24)	Very toxic
TCPP	13674-84-5	Flame retardant (plastic additive)	1600	2.59	Water stock	> 5000	n.c.	–
					DMSO stock	> 5000	n.c.	–
Sulfamethoxazole (SFX)	723-46-6	Pharmaceutical	610	0.89	DMSO stock	5000	6134 (4615–11,873)	Hazardous
Ibuprofen (IBU)	15687-27-1	Pharmaceutical	21	3.97	DMSO stock	> 10,000	n.c.	–
TDCPP	13674-87-8	Flame retardant (plastic additive)	18.1	3.69	DMSO stock	> 5000	n.c.	–
Galaxolide (HHCB)	122-05-5	Cosmetic fragrance	1.75	5.9	DMSO stock	500	733 (434–1032)	Toxic
4-MBC	36861-47-9	Cosmetic UV filter	0.2–17	4.95–5.92	DMSO stock	600 ^(*)^	239 (150–343)^(*)^	Very toxic
Octocrylene (OCT)	6197-30-4	Cosmetic UV filter	0.36	6.88	DMSO stock	–	162 (30–270)	Very toxic
Chimassorb (CHI)	192268-64-7	UV stabilizer (plastic additive)	0.2	–	DMSO stock	> 300	n.c.	–
					Lixiviate	> 1* 10^5^	n.c.	Non-toxic
BHT	128-37-0	Antioxidant (plastic additive)	0.4	5.1	DMSO stock	100	91 (57–121)	Very toxic
					Lixiviate	1* 10^5^	3.6*10^4^ (n.c.)	Non-toxic
Lawsone (LAW)	83-72-7	Antimicrobial	0.2–< 1000	1.38–4.1^a^	DMSO stock	9000	13,821 (9614–20,904)	Non-toxic
					Lixiviate	33333	24,636 (n.c.)	Non-toxic

The substances were tested by means of serial dilutions of a stock dissolved in ultrapure water, a stock dissolved in DMSO, or a 0.1 g/L lixiviate. Lowest observed effect concentration (LOEC) for BAHC. EC_10_ for all compounds and 95% confidence intervals given in brackets. Lowest observed effect concentration (LOEC) levels for all the compounds. In the table, n.c. means “not calculated”. Water solubility and *K*_ow_ data were obtained from https://pubchem.ncbi.nlm.nih.gov/ except when otherwise stated.

*Data from Paredes et al. (2014)

^a^From: https://www.parchem.com/chemical-supplier-distributor/Lawsone-042337.aspx

For chemicals with a *S*_w_ < 1 mg/L, in addition to the DMSO test, a leachate test was conducted. With that aim, a leachate was obtained using ASW as liquid phase, and serial dilutions were further tested according to the procedures of Tier I in Beiras et al. (2019). The lixiviate was separated from the insoluble chemical by filtering with GF/F (Whatman, Merck, nominal pore 0.7 µm) filters for solid substances and by centrifugation at 2000 rpm for 5 min for liquid substances. The solid-to-liquid ratio used for preparation of lixiviates was 100 mg/L, the level below which current EU legislation consider a substance as not harmful to aquatic life (European Commission, 2011). In order to assess the effect of the chemical to ASW ratio in the lixiviate on toxicity classification, additional experiments were conducted comparing loads from 0.01 to 10 g/L using BHT and CHI as model substances.

### Statistical methods

Statistical analyses were conducted using IBM SPSS statistics software (version 25.0). Larval size for sea urchin and survival for copepod larvae were the endpoints recorded. All data were corrected by the mean control response. Normal distribution and homoscedasticity of the data were checked using the Shapiro–Wilk’s and Levene’s tests, respectively. When significant differences (*p* < 0.05) among groups were found using ANOVA, then each treatment was compared to the control using Dunnett’s post-hoc test to calculate the highest no-observed effect concentration (NOEC) and the lowest observed effect concentration (LOEC). Non-parametric tests, Kruskall-Wallis, and Mann Whitney U were used when data did not meet the requirements for parametric tests. The EC_50_ and EC_10_ values and their 95% confidence intervals were calculated by using a Probit non-linear regression model. In lixiviate tests, toxic units (TU) were defined as the dilution factor of the lixiviate that causes a 50% effect on the endpoint. The sensitivity of the two biological models (sea urchins and copepods) to BAHCs was compared by fitting the EC_50_ values to a linear correlation model and testing whether the slope is significantly different to 1.

## Results and discussion

### Toxicity tests using stock dilutions

As reflected in Table 1, the most toxic BAHC tested was FLU (EC_10_ = 14 µg/L, 95% confidence interval 6–24) followed by BHT (EC_10_ = 91 µg/L, 57–121), OCT (EC_10_ = 162 µg/L, 567–1091), and 4-MBC (EC_10_ = 239 µg/L, 150–343). These chemicals can be classified as very toxic to aquatic life according to Regulation No 286/2011 (European Commission, 2011), since their EC_50_ was lower than 1 mg/L (see Table 1). The ranking of toxicity decreases with HHCB (EC_10_ = 733 µg/L, 434–1032), which can be classified as toxic (EC_50_ = 1276 µg/L, 1065–1486), SUL (EC_10_ = 6.1 mg/L, 4.6–11.8), and LAW (EC_10_ = 13.8 mg/L, 9.6–20.9). IBU and the plastic additives TDCP, TCPP, and CHI were the least toxic chemicals tested.

The *A. clausi* test yielded a slightly different ranking, with toxicity decreasing in the order OCT (EC_10_ = 13 µg/L, 95% confidence interval not calculable, n.c.), FLU (EC_10_ = 34 µg/L, n.c.), 4-MBC (EC_10_ = 39 µg/L, 4–76), BHT (EC_10_ = 78 µg/L. 8–117), TDCP (EC_10_ = 126), and LAW (EC_10_ = 1909 µg/L, n.c.). Therefore, sea urchin larvae were more sensitive to FLU than *A. clausi* nauplii, whereas OCT, 4-MBC, TDCP, and LAW affected more the copepod than the sea-urchin larvae. However, it is remarkable that both tests produce consistent classifications; OCT, FLU, 4-MBC, and BHT, when tested with *Acartia*, would be classified again as very toxic, whereas LAW would be classified just as hazardous to aquatic life. In contrast, TDCP seems to have a more specific mode of action affecting crustaceans but not echinoderms.

When results from laboratory toxicity tests using single substances in chemically defined water are used to derive environmental standards, we must take into account the potential effects of humic substances with the ability to complex toxic molecules, as well as the potential interactions among chemicals that may take place in more complex environmental scenarios.

### Comparison of stock dilution vs lixiviates

**Fig. 1:**
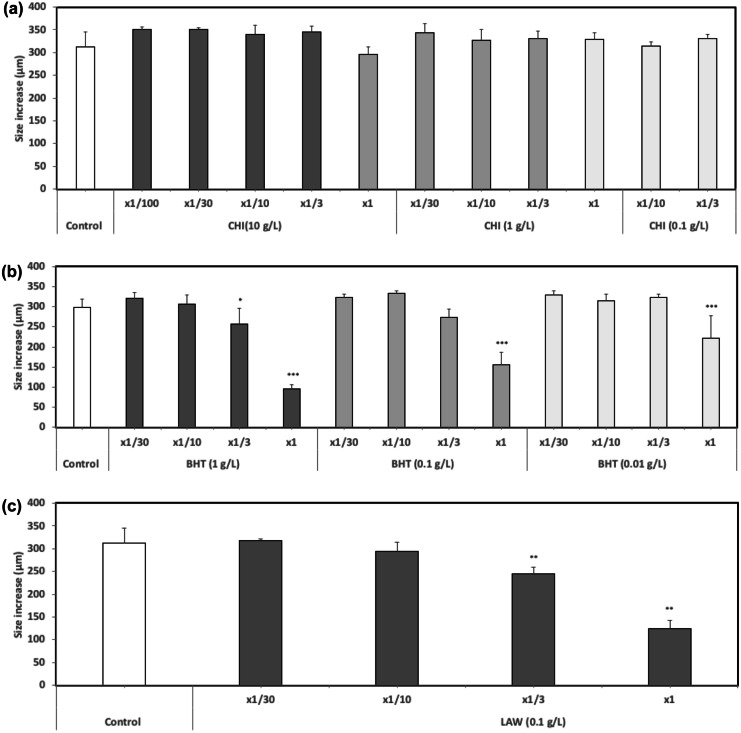
*P. lividus* larval size increases, in serial dilutions (× 1/30, × 1/10, × 1/3, × 1) of CHI (**a**), BHT (**b**), and LAW (**c**) leachates in ASW. The different solid-to-liquid ratios (g/L) used to make up the leachates are indicated. Bars represent mean ± SD (*N* = 4). Asterisks refer to significant differences to control (Control) *p < 0.05, **p < 0.01 and ***p < 0.001

For highly hydrophobic chemicals (*S*_w_ < 1 mg/L), the toxicity obtained from testing serial dilutions of the DMSO stock was compared with that of lixiviates obtained by mixing the substance in ASW at a 100 mg per litter solid-to-liquid ratio for 24 h (Table 1). For the antimicrobial LAW, larval size increased as the lixiviate was diluted according to a dose:response pattern (Fig. 1c) from which a TU value of 1.23 (0.92–1.53) was obtained. This would correspond to an EC_50_ value of 100/1.23 = 81.3 mg/L. In the DMSO stock test, the EC_10_ was 13.8 mg/L but the EC_50_ could not be calculated since growth inhibition was lower than 50% at the higher concentration tested. However, a rough estimation of the EC_50_ can be obtained from the average EC_50_/EC_10_ ratio of 3.3 compiled from similar tests by Durán and Beiras (2017). This value is 45.6 mg/L, within the same order of magnitude than that obtained from the lixiviate test (81.3 mg/L), and in both cases classifying LAW within the category of substances hazardous to aquatic life (10 < EC_50_ ≤ 100 mg/L).

In contrast, for BHT, large discrepancies in toxicity classification were obtained depending on the method of dosage, with the lixiviate method failing to detect any toxicity (EC_50_ > 100 mg/L) whereas the DMSO stock method classifies this substance as very toxic disregarding the test species used. The role of the organic carrier as vehicle of BHT seems to largely enhance the toxicity of this highly hydrophobic substance compared to exposure to the aqueous lixiviate. While the precautionary principle would support the use of the DMSO stock method, a more environmentally realistic estimation of risk would opt for the lixiviate method (see next section also).

### Solid-to-liquid ratio for lixiviation

For the highly hydrophobic chemicals CHI and BHT, the toxicity of leachates obtained using different solid-to-liquid ratios, from 0.01 to 10 g/L in × 10 geometric sequence was compared using the sea-urchin test (Fig. 1). CHI (Fig. 1a) was consistently classified as non-toxic regardless the ratio used, although the lowest larval size was recorded in the undiluted leachate with the highest solid-to-liquid ratio, 10 g/L. On the other hand, BHT (Fig. 1b) showed toxicity, i.e., caused a significant decrease in larval size, in all undiluted leachates tested.

However, once toxicity is expressed in TU, no comparisons can be made among lixiviates obtained using different solid-to-liquid ratio. As expected from the low solubility of the chemical, higher amounts of substance used to make up the lixiviate fail to achieve a proportional increase in the lixiviate toxicity, expressed in TU. A tenfold increase in g/L produced on average just a 1.4-fold increase in TU.

Therefore, despite working with chemical loads orders of magnitude above their theoretical water solubility, lixiviates obtained at higher solid-to-liquid ratios were more toxic to the larvae. This suggests that part of the non-dissolved chemical is accommodated into the liquid phase and becomes partly bioavailable when the lixiviate is diluted.

These differences in toxicity among lixiviates obtained at different solid-to-liquid ratios stress the need for methodological standardization. Toxicity data can, thus, be compared only when lixiviation is conducted using the same solid-to-liquid ratio. Lower ratios decrease the limit of detection of toxicity, whereas higher ratios may underestimate TU values, require high amounts of pure chemicals, and produce more toxic waste. A solid-to-liquid ratio of 0.1 g/L, i.e., 100 mg/L, seems to balance sensitivity, environmental relevance, and feasibility, and allows results to be useful for classification of substances according to their aquatic toxicity, since 100 mg/L is the EC_50_ threshold above which substances are considered as not hazardous.

### Comparison among aquatic test species

While metals, polyaromatic hydrocarbons, and some pesticides are regulated and water quality standards are available world-wide, BAHC lack environmental standards, which can only be implemented on the basis of sound ecotoxicological information using standardized procedures (Beiras & Schönemann, 2021, and citations therein). Table S1 compares the toxicity thresholds found in the present study with those previously reported for other marine test species. This information provides the grounds for derivation of marine water quality criteria for these emerging pollutants, currently not included in environmental regulations. The toxicity found with sea-urchin larvae is also similar to acute toxicity reported for the standard freshwater model *Daphnia*. For example, HHCB showed on *P. lividus* an EC_50_ = 1276 μg/L, similar to that previously reported on *D. magna* with a 48 h-LC_50_ of 1170 μg/L (Chen et al., 2015) and 2684 μg/L (Fan et al., 2019).

**Fig. 2:**
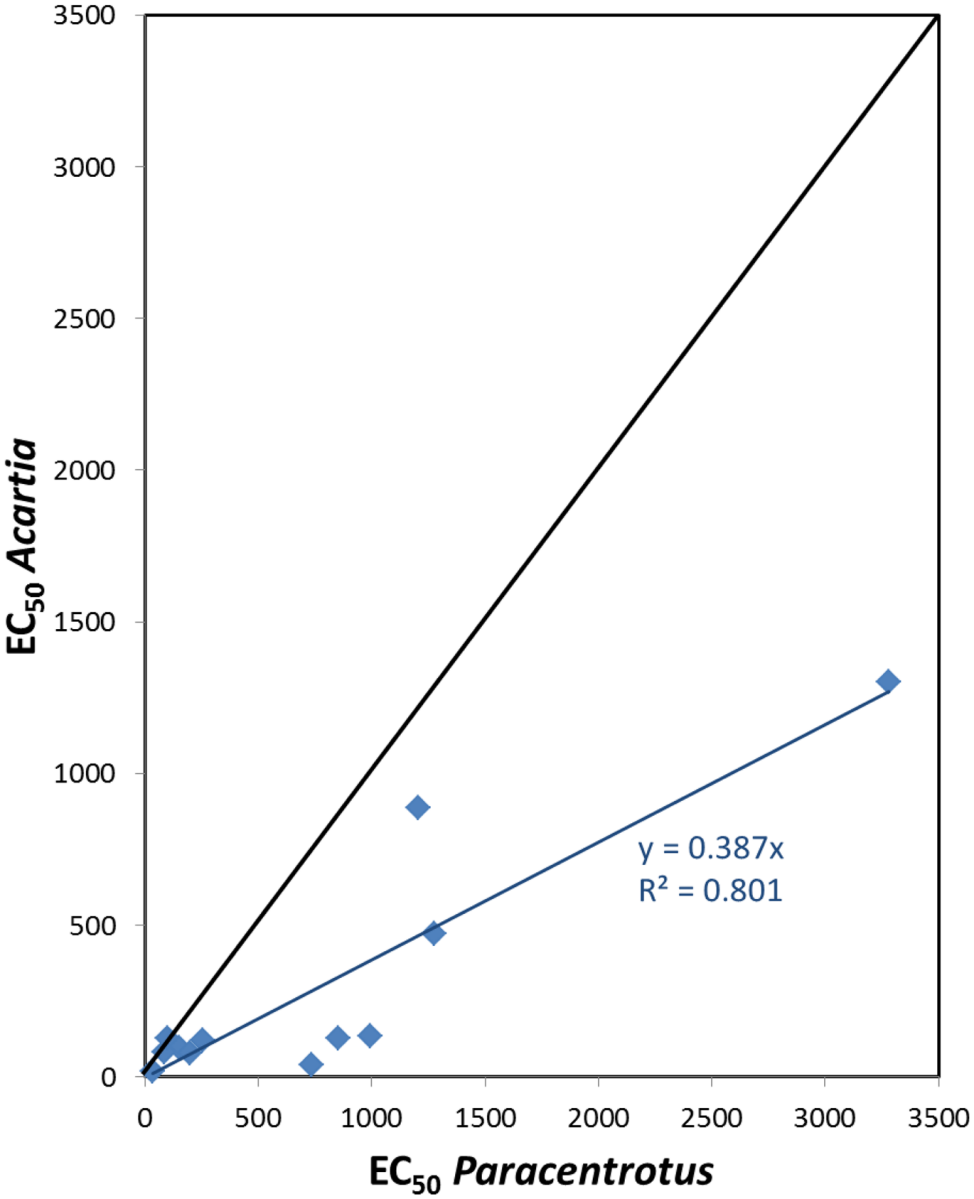
EC_50_ values (µg/L) of different chemicals recorded in 48-h tests using larvae of *Acartia spp* and *Paracentrotus lividus* (Table S2). Notice that *Acartia* values are lower, indicating a higher sensitivity, reflected also in the slope < 1 of the linear correlation equation

In contrast, the nauplius larvae of the marine copepod *Acartia* seems to be more sensitive than other aquatic test species. Figure 2 compares the sensitivity to organic and metallic toxicants, in terms of 48-h EC_50_, between sea urchin and *Acartia* larvae. For 11 of the 12 chemicals reviewed, the Acartia EC_50_ value was lower, which reflects in a slope significantly lower than 1 in the equation of the linear correlation *Acartia* EC_50_ vs *Paracentrotus* EC_50_ (b = 0.39; 95% CI 0.26–0.53).

*Acartia* nauplii seem also more sensitive than the standard freshwater model *Daphnia*. Fent et al. (2010) found an acute LC_50_ for 4-MBC in *D. magna* of 560 μg/L, whereas in our experiment, *A. clausi* showed a LC_50_ of 127 μg/L. Nielsen and Roslev (2018) reported an EC_50_ value of 2290 μg/L of FLU on *D. magna* mobility, remarkably higher that *Acartia*’s LC_50_ of 128 μg/L. Wollenberger et al. (2003) reported for *Acartia tonsa* exposed to HCCB an EC_10_ of 37 µg/L markedly lower than Daphnia’s LOEC [205 µg/L, cited by Balk and Ford (1999)].

## Conclusions

Common everyday household chemicals such the antioxidant BHT, the UV filters broadly present in cosmetics OCT and 4-MBC, or the pharmaceutical FLU, are consistently classified as very toxic to aquatic life according to their toxicity to early life stages of marine invertebrates. These findings stress the need to develop water quality criteria for these chemicals on the basis of standard toxicity testing procedures. We provide a protocol for testing based on water solubility and *K*_ow_ in order to achieve comparable toxicity parameters useful for the classification of household chemicals and derivation of environmental quality criteria and standards for these emerging pollutants. The use of *Acartia* nauplii in this protocol provides high sensitivity and, thus, protective value to the toxicity values obtained according to the present protocol.

## Supplementary information

Below is the link to the electronic supplementary material.

Supplementary file1 (DOCX 258 kb)

## Data Availability

Data, associated meta-data, and calculation tools are available from the corresponding author (rbeiras@uvigo.gal).
