# Supplementary material for: Towards standard methods for the classification of aquatic toxicity for biologically active household chemicals (BAHC) present in plastics, pharmaceuticals, and cosmetic products
Source: Environ Monit Assess. 2021 Oct 2;193(10):685. doi: 10.1007/s10661-021-09436-w (PMC8487416; doi:10.1007/s10661-021-09436-w)
Supplement: Supplementary file 1 — Supplementary file1 (DOCX 258 kb) [file 10661_2021_9436_MOESM1_ESM.docx]

*Figure S1.* Flow chart with the decision tree used to choose the method of dosing in the toxicity tests. The chemical properties used were water solubility and octanol-water partition coefficient (Kow), both widely available for most chemicals. Artificial sea water (ASW), Solubility in water (Sw), Solubility in octanol (S oct).


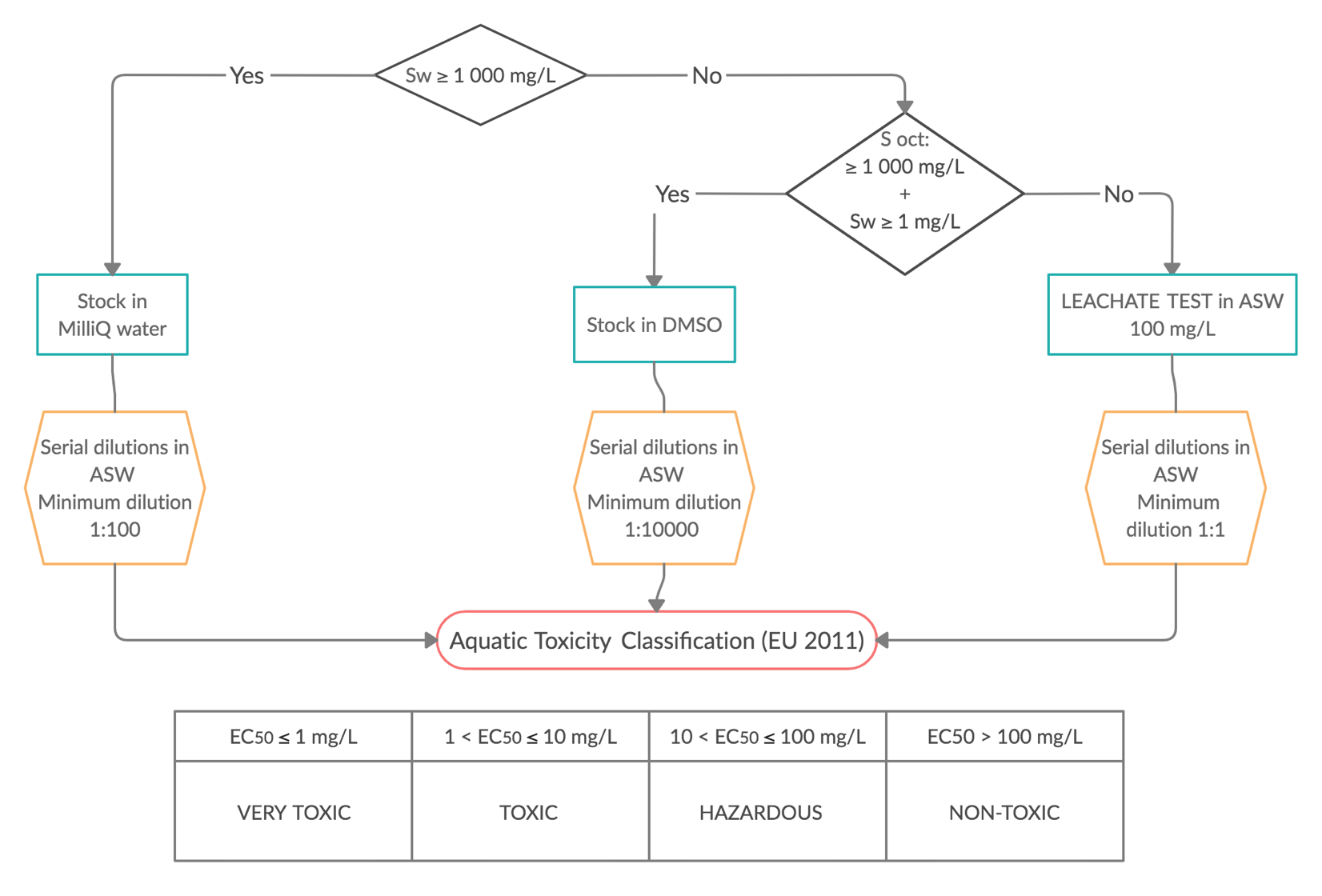


*Table S1.* Review of toxicity thresholds (TT) reported in marine species for the BAHC tested in the present study. Values are marked in bold when source indicates standard deviation or confidence intervals.

| **Chemical** | **Taxon** | **Species** | **Endpoint** | **TT** | **µg L^-1^** | **Reference** |
| --- | --- | --- | --- | --- | --- | --- |
| **Sulphamethoxazole** | Algae | *Nannochloropsis oculata* | Cell density (20 d) | LOEC | 0.0075 | ([Teixeira and Granek 2017](#_ENREF_23)) |
|  |  | *Chaetoceros neogracile* | Cell density (20 d) | LOEC | 0.075 | ([Teixeira and Granek 2017](#_ENREF_23)) |
|  | Rotifers | *Brachionus koreanus* | Survival (24 h) | NOEC | 197,500 | ([Rhee et al. 2012](#_ENREF_21)) |
|  | Echinoderms | *Paracentrotus lividus* | Early larval growth (48 h) | EC_10_ | **6134** | This study |
| **Ibuprofen** | Algae | *Isochrysis galbana* | Populat. growth (72 h) | EC_10_ | **22,600** | ([Aguirre-Martínez et al. 2015](#_ENREF_2)) |
|  |  | *Skeletonema costatum* | Populat. growth (96 h) | EC_50_/3 | 2367 | Cited by ([Webb 2004](#_ENREF_25)) |
|  | Mollusks | *Mytilus galloprovincialis* | Larval morphology (48 h) | LOEC | 100 | ([Fabbri et al. 2014](#_ENREF_14)) |
|  |  | *Mytilus trossulus* | Energy balance (14 d) | LOEC | 1000 | ([Ericson et al. 2010](#_ENREF_13)) |
|  | Crustaceans | *Carcinus maenas* | Lysosomal estability (28 d) | LOEC | 5 | ([Aguirre-Martínez et al. 2013](#_ENREF_1)) |
|  | Echinoderms | *P. lividus* | Larval morphology (48 h) | LOEC | 0.01 ^(1)^ | ([Aguirre-Martínez et al. 2015](#_ENREF_2)) |
|  |  | *P. lividus* | Early larval growth (48 h) | LOEC | >10,000 | This study |
|  |  | *Psammechinus miliaris* | Fertilization success (60 min) | EC_50_/3 | 264 | ([Zanuri et al. 2017](#_ENREF_28)) |
| **Fluoxetine** | Algae | *Dunaliella tertiolecta* | Population growth (96 h) | EC_50_/3 | **56.6** | ([DeLorenzo and Fleming 2008](#_ENREF_11)) |
|  |  | *Dunaliella salina* | Population growth (96 h) | LOEC | 46.4 | ([Bi et al. 2018](#_ENREF_7)) |
|  |  | *Dunaliella parva* | Population growth (96 h) | LOEC | 18.6 | ([Bi et al. 2018](#_ENREF_7)) |
|  |  | *S. costatum* | Population growth (72 h) | EC_50_/3 | **5.4** | ([Petersen et al. 2014](#_ENREF_20)) |
|  |  | *Skeletonema marinoi* | Population growth (72 h) | EC_10_ | 7.3 | ([Minguez et al. 2018](#_ENREF_18)) |
|  |  | Marine periphyton | Total pigment content (96 h) | EC_50_/3 | **12** | ([Backhaus et al. 2011](#_ENREF_3)) |
|  | Mollusks | *Crassostrea gigas* | Embryogenesis (36 h) | EC_50_/3 | 63.8 | ([Di Poi et al. 2014](#_ENREF_12)) |
|  |  | *C. gigas* | Metamorphosis (24 h) | EC_50_/3 | 62.6 | ([Di Poi et al. 2014](#_ENREF_12)) |
|  |  | *Chlorostoma funebralis* | Foot detachment (4 h) | LOEC | 345 | ([Fong and Molnar 2013](#_ENREF_15)) |
|  | Crustaceans | *Acartia clausi* | Larval mortality (48 h) | LC_10_ | 34.4 | This study |
|  | Echinoderms | *P. lividus* | Early larval growth (48 h) | EC10 | 14.0 | This study. |
|  | Fish | *Cyprinodon variegatus* | Locomotion decrease (56 h) | EC_25_ | **104** | ([Winder et al. 2012](#_ENREF_26)) |
|  |  | *Menidia beryllina* | Aduly mortality (48 h) | LC_50_/3 | 167 | ([Daigle 2010](#_ENREF_10)) |
| **4-MBC** | Algae | *I. galbana* | Population growth (72 h) | EC_10_ | **5.4** | ([Paredes et al. 2014](#_ENREF_19)) |
|  | Mollusk | *M. galloprovincialis* | Early larval growth (48 h) | EC_10_ | **411** | ([Paredes et al. 2014](#_ENREF_19)) |
|  | Crustaceans | *Siriella armata* | Neonate mortality (96 h) | LC_10_ | **71.6** | ([Paredes et al. 2014](#_ENREF_19)) |
|  |  | *Tigriopus japonicus* | Immobilization (72 h) | LOEC | 10 | ([Chen et al. 2018](#_ENREF_9)) |
|  |  | *T. japonicus* | Reproduction (4 generations) | LOEC | 0.5 | ([Chen et al. 2018](#_ENREF_9)) |
|  |  | *A. clausi* | Larval mortality (48 h) | EC_10_ | **38.9** | This study |
|  | Echinoderms | *P. lividus* | Early larval growth (48 h) | EC_10_ | **239** | ([Paredes et al. 2014](#_ENREF_19)) |
|  |  | *P. lividus* | Larval growth (48 h) | LOEC | 2 | ([Torres et al. 2016](#_ENREF_24)) |
| **Octocrylene** | Algae | *I. galbana* | Population growth (72 h) | EC_10_ | **103** | ([Giraldo et al. 2017](#_ENREF_16)) |
|  | Mollusk | *M. galloprovincialis* | Larval development (48 h) | EC_10_ | **511** | ([Giraldo et al. 2017](#_ENREF_16)) |
|  | Crustaceans | *A. clausi* | Larval mortality (48 h) | LOEC | **50** | This study |
|  | Echinoderms | *P. lividus* | Early larval growth (48 h) | EC_10_ | **162** | ([Giraldo et al. 2017](#_ENREF_16)) |
|  |  | *Strongylocentrotus purpuratus* | Larval morphology (96 h) | EC_50_/3 | **0.087** | ([Giraldo et al. 2017](#_ENREF_16)) |
|  | Fish | *C. variegatus* | Hatching (9 d) | LOEC | 960 | Beiras & Veiga, unpublished |
| **Galaxolide** | Crustaceans | *Nitokra spinipes* | Adult mortality (96 h) | LC_50_/3 | 633 | ([Breitholtz et al. 2003](#_ENREF_8)) |
|  |  | *N. spinipes* | Larval development (7-8 d) | LOEC | 20 | ([Breitholtz et al. 2003](#_ENREF_8)) |
|  |  | *Acartia tonsa* | Larval development (5 d) | EC_10_ | 37 | ([Wollenberger et al. 2003](#_ENREF_27)) |
|  |  | *A. tonsa* | Adult mortality (48 h) | LC_10_ | 120 | ([Wollenberger et al. 2003](#_ENREF_27)) |
|  | Echinoderms | *P. lividus* | Early larval growth (48 h) | LOEC | 500 | This study |

^(1)^ Non-monotonic response, with effects disappearing at higher concentrations

*Table S2.* Review of the 48 h- EC_50_ values for different BAHCs reported in *Acartia sp* nauplius survival and *P. lividus* embryo*-*larval development*.*

|  | **Species** | **EC_50_ (µg/L)** | **Reference** |  |
| --- | --- | --- | --- | --- |
| **Triclosan** | *Acartia sp.* | 94.3 | ([Tato et al. 2018](#_ENREF_22)) |  |
|  | *Paracentrotus lividus* | 149.3 | ([Tato et al. 2018](#_ENREF_22)) |  |
| **Bisphenol-A** | *Acartia sp.* | 885 | ([Tato et al. 2018](#_ENREF_22)) |  |
|  | *P. lividus* | 1207 | ([Tato et al. 2018](#_ENREF_22)) |  |
| **branched 4-NP** | *Acartia sp.* | 78.5 | Beiras & Fariña, unpublished |  |
|  | *P. lividus* | 200.7 | Beiras & Fariña, unpublished |  |
| **linear 4-n-NP** | *Acartia sp.* | 82.7 | ([Tato et al. 2018](#_ENREF_22)) | |
|  | *P. lividus* | 84.7 | ([Tato et al. 2018](#_ENREF_22)) | |
| **Benzophenone-3** | *Acartia sp.* | 1302 | ([Paredes et al. 2014](#_ENREF_19)) | |
|  | *P. lividus* | 3280 | ([Paredes et al. 2014](#_ENREF_19)) | |
| **4-MBC** | *Acartia sp.* | 127 | This study | |
|  | *P. lividus* | 854 | ([Paredes et al. 2014](#_ENREF_19)) | |
| **Octochrylene** | *Acartia sp.* | 40.3 | This study | |
|  | *P. lividus* | 737 | (Giraldo et al. 2017) | |
| **Galaxolide** | *Acartia sp.* | 470 | ([Wollenberger et al. 2003](#_ENREF_27)) | |
|  | *P. lividus* | 1276 | This study | |
| **Butyl-hydroxytoluene** | *Acartia sp.* | 133.5 | This study | |
|  | *P. lividus* | 995 | This study | |
| **Cu** | *Acartia sp.* | 19 | Beiras & Vilas, unpublished | |
|  | *P. lividus* | 32.9 | ([Lorenzo et al. 2002](#_ENREF_17)) | |
| **Fluoranthene** | *Acartia sp.* | 120 | ([Bellas and Thor 2007](#_ENREF_6)) | |
|  | *P. lividus* | 253 | ([Bellas et al. 2008](#_ENREF_5)) | |
| **Fluoxetine** | *Acartia sp.* | 128 | This study | |
|  | *P. lividus* | 97.5 | This study | |
| **TCPP** | *Acartia sp.* | >1250 | This study | |
|  | *P. lividus* | >5000 | This study | |
| **TDCPP** | *Acartia sp.* | 601 | Beiras & López Ibáñez, unpublished | |
|  | *P. lividus* | >5000 | This study | |
| **Acetylated monoglyceride** | *Acartia sp.* | >100,000 | Beiras & Campoy-López, unpublished | |
|  | *P. lividus* | >100,000 | (Beiras et al. 2021) | |
| **Tocopherol** | *Acartia sp.* | >100,000 | Beiras & Campoy-López, unpublished | |
|  | *P. lividus* | >100,000 | (Beiras et al. 2021) | |
| **Quercetin** | *Acartia sp.* | 14,556 | Beiras & Vilas, unpublished | |
|  | *P. lividus* | 36,200 | (Beiras et al. 2021) | |

**SUPPLEMENTARY REFERENCES.**

Aguirre-Martínez, G., S. Buratti, E. Fabbri, T. Del Valls and M. Martín-Díaz. 2013. Stability of lysosomal membrane in Carcinus maenas acts as a biomarker of exposure to pharmaceuticals. *Environmental monitoring and assessment*, 185 (5): 3783-3793.

Aguirre-Martínez, G. V., A. T. DelValls and M. L. Martín-Díaz. 2015. Yes, caffeine, ibuprofen, carbamazepine, novobiocin and tamoxifen have an effect on Corbicula fluminea (Müller, 1774). *Ecotoxicology and environmental safety*, 120: 142-154.

Backhaus, T., T. Porsbring, Å. Arrhenius, S. Brosche, P. Johansson and H. Blanck. 2011. Single‐substance and mixture toxicity of five pharmaceuticals and personal care products to marine periphyton communities. *Environmental toxicology and chemistry*, 30 (9): 2030-2040.

Beiras, R., Verdejo, E., Campoy-López, P., Vidal-Liñán, L. 2021. Aquatic toxicity of chemically defined microplastics can be explained by functional additives. *Journal of Hazardous Materials*, 406: 124338.

Bellas, J., L. Saco-Álvarez, Ó. Nieto and R. Beiras. 2008. Ecotoxicological evaluation of polycyclic aromatic hydrocarbons using marine invertebrate embryo–larval bioassays. *Marine pollution bulletin*, 57 (6-12): 493-502.

Bellas, J. and P. Thor. 2007. Effects of selected PAHs on reproduction and survival of the calanoid copepod Acartia tonsa. *Ecotoxicology*, 16 (6): 465-474.

Bi, R., X. Zeng, L. Mu, L. Hou, W. Liu, P. Li, H. Chen, D. Li, A. Bouchez and J. Tang. 2018. Sensitivities of seven algal species to triclosan, fluoxetine and their mixtures. *Scientific reports*, 8 (1): 1-10.

Breitholtz, M., L. Wollenberger and L. Dinan. 2003. Effects of four synthetic musks on the life cycle of the harpacticoid copepod Nitocra spinipes. *Aquatic Toxicology*, 63 (2): 103-118.

Chen, L., X. Li, H. Hong and D. Shi. 2018. Multigenerational effects of 4-methylbenzylidene camphor (4-MBC) on the survival, development and reproduction of the marine copepod Tigriopus japonicus. *Aquatic Toxicology*, 194: 94-102.

Daigle, J. K. 2010. Acute responses of freshwater and marine species to ethinyl estradiol and fluoxetine.

DeLorenzo, M. E. and J. Fleming. 2008. Individual and mixture effects of selected pharmaceuticals and personal care products on the marine phytoplankton species Dunaliella tertiolecta. *Archives of Environmental Contamination and Toxicology*, 54 (2): 203-210.

Di Poi, C., L. Evariste, A. Serpentini, M.-P. Halm-Lemeille, J.-M. Lebel and K. Costil. 2014. Toxicity of five antidepressant drugs on embryo–larval development and metamorphosis success in the Pacific oyster, Crassostrea gigas. *Environmental Science and Pollution Research*, 21 (23): 13302-13314.

Ericson, H., G. Thorsén and L. Kumblad. 2010. Physiological effects of diclofenac, ibuprofen and propranolol on Baltic Sea blue mussels. *Aquatic Toxicology*, 99 (2): 223-231.

Fabbri, R., M. Montagna, T. Balbi, E. Raffo, F. Palumbo and L. Canesi. 2014. Adaptation of the bivalve embryotoxicity assay for the high throughput screening of emerging contaminants in Mytilus galloprovincialis. *Marine environmental research*, 99: 1-8.

Fong, P. P. and N. Molnar. 2013. Antidepressants cause foot detachment from substrate in five species of marine snail. *Marine environmental research*, 84: 24-30.

Giraldo, A., R. Montes, R. Rodil, J. Quintana, L. Vidal-Liñán and R. Beiras. 2017. Ecotoxicological evaluation of the UV filters ethylhexyl dimethyl p-aminobenzoic acid and octocrylene using marine organisms *Isochrysis galbana*, *Mytilus galloprovincialis* and *Paracentrotus lividus*. *Archives of Environmental Contamination and Toxicology*, 72 (4): 606-611.

Lorenzo, J., O. Nieto and R. Beiras. 2002. Effect of humic acids on speciation and toxicity of copper to Paracentrotus lividus larvae in seawater. *Aquatic Toxicology*, 58 (1-2): 27-41.

Minguez, L., R. Bureau and M.-P. Halm-Lemeille. 2018. Joint effects of nine antidepressants on Raphidocelis subcapitata and Skeletonema marinoi: A matter of amine functional groups. *Aquatic Toxicology*, 196: 117-123.

Paredes, E., S. Perez, R. Rodil, J. Quintana and R. Beiras. 2014. Ecotoxicological evaluation of four UV filters using marine organisms from different trophic levels Isochrysis galbana, *Mytilus galloprovincialis*, *Paracentrotus lividus*, and *Siriella armata*. *Chemosphere*, 104: 44-50.

Petersen, K., H. H. Heiaas and K. E. Tollefsen. 2014. Combined effects of pharmaceuticals, personal care products, biocides and organic contaminants on the growth of Skeletonema pseudocostatum. *Aquatic Toxicology*, 150: 45-54.

Rhee, J.-S., C.-B. Jeong, B.-M. Kim and J.-S. Lee. 2012. P-glycoprotein (P-gp) in the monogonont rotifer, *Brachionus koreanus*: Molecular characterization and expression in response to pharmaceuticals. *Aquatic Toxicology*, 114: 104-118.

Tato, T., N. Salgueiro-González, V. M. León, S. González and R. Beiras. 2018. Ecotoxicological evaluation of the risk posed by bisphenol A, triclosan, and 4-nonylphenol in coastal waters using early life stages of marine organisms (*Isochrysis galbana, Mytilus galloprovincialis, Paracentrotus lividus*, and *Acartia clausi*). *Environmental Pollution*, 232: 173-182.

Teixeira, J. R. and E. F. Granek. 2017. Effects of environmentally-relevant antibiotic mixtures on marine microalgal growth. *Science of the Total Environment*, 580: 43-49.

Torres, T., I. Cunha, R. Martins and M. M. Santos. 2016. Screening the toxicity of selected personal care products using embryo bioassays: 4-MBC, propylparaben and triclocarban. *International journal of molecular sciences*, 17 (10): 1762.

Webb, S. 2004. A Data Based Perspective on the Environmental Risk Assessment of Human Pharmaceuticals Il—Aquatic Risk Characterisation. Pharmaceuticals in the environment. Springer. 345-361.

Winder, V. L., P. L. Pennington, M. W. Hurd and E. F. Wirth. 2012. Fluoxetine effects on sheepshead minnow (Cyprinodon variegatus) locomotor activity. *Journal of Environmental Science and Health, Part B*, 47 (1): 51-58.

Wollenberger, L., M. Breitholtz, K. O. Kusk and B.-E. Bengtsson. 2003. Inhibition of larval development of the marine copepod Acartia tonsa by four synthetic musk substances. *Science of the Total Environment*, 305 (1-3): 53-64.

Zanuri, N. B. M., M. G. Bentley and G. S. Caldwell. 2017. Assessing the impact of diclofenac, ibuprofen and sildenafil citrate (Viagra®) on the fertilisation biology of broadcast spawning marine invertebrates. *Marine environmental research*, 127: 126-136.
